# The promising shadow of microbubble over medical sciences: from fighting wide scope of prevalence disease to cancer eradication

**DOI:** 10.1186/s12929-021-00744-4

**Published:** 2021-06-21

**Authors:** Ali Jangjou, Amir Hossein Meisami, Kazem Jamali, Mohammad Hadi Niakan, Milad Abbasi, Mostafa Shafiee, Majid Salehi, Ahmad Hosseinzadeh, Ali Mohammad Amani, Ahmad Vaez

**Affiliations:** 1 Department of Emergency Medicine, School of Medicine, Shiraz University of Medical Sciences, Shiraz, Iran; 2 Department of Emergency Medicine, School of Medicine, Kermanshah University of Medical Sciences, Kermanshah, Iran; 3 Trauma Research Center, Shahid Rajaee (Emtiaz) Trauma Hospital, Shiraz University of Medical Sciences, Shiraz, Iran; 4 Department of Medical Nanotechnology, School of Advanced Medical Sciences and Technologies, Shiraz University of Medical Sciences, Shiraz, Iran; 5 Department of Tissue Engineering, School of Medicine, Shahroud University of Medical Sciences, Shahroud, Iran; 6 Tissue Engineering and Stem Cells Research Center, Shahroud University of Medical Sciences, Shahroud, Iran; 7 Thoracic and Vascular Surgery Research Center, Nemazee Hospital, Shiraz University of Medical Sciences, Shiraz, Iran; 8 Department of Tissue Engineering and Applied Cell Sciences, School of Advanced Medical Sciences and Technologies, Shiraz University of Medical Sciences, Shiraz, Iran

**Keywords:** Microbubbles, Biomedicine, Drug delivery, Cancer treatment, Nanoparticles

## Abstract

Microbubbles are typically 0.5–10 μm in size. Their size tends to make it easier for medication delivery mechanisms to navigate the body by allowing them to be swallowed more easily. The gas included in the microbubble is surrounded by a membrane that may consist of biocompatible biopolymers, polymers, surfactants, proteins, lipids, or a combination thereof. One of the most effective implementation techniques for tiny bubbles is to apply them as a drug carrier that has the potential to activate ultrasound (US); this allows the drug to be released by US. Microbubbles are often designed to preserve and secure medicines or substances before they have reached a certain area of concern and, finally, US is used to disintegrate microbubbles, triggering site-specific leakage/release of biologically active drugs. They have excellent therapeutic potential in a wide range of common diseases. In this article, we discussed microbubbles and their advantageous medicinal uses in the treatment of certain prevalent disorders, including Parkinson's disease, Alzheimer's disease, cardiovascular disease, diabetic condition, renal defects, and finally, their use in the treatment of various forms of cancer as well as their incorporation with nanoparticles. Using microbubble technology as a novel carrier, the ability to prevent and eradicate prevalent diseases has strengthened the promise of effective care to improve patient well-being and life expectancy.

## Introduction

A microbubble is called a technological structure that interacts dynamically with the vital organs of the body at the cellular stage [1–3]. These non-toxic and biocompatible structures should have an outer layer with an average dimension of 0.1–10 µm (less than 50 µm), a limited range of measurements to avoid complications [4, 5]. Microbubbles should also have a thickness and compressive strength gap between themselves and the underlying body tissues in order to generate acoustic impedance and disperse the US in an amount greater than the amount of body tissue to be known as a contrast agent [6–8]. Also, the microbubbles must have the appropriate chemical surface properties to not only bind by different ligands to different organs and tissues but also to preserve the thickness of the shell of the microbubble [9]. The diameter of the microbubbles, their material content in their shell layer, may significantly affect the longevity and acoustic behavior of the microbubbles [10]. Some of microbubbles, made up of air or oxygen, are capable of being suspended inside the water for a very long time. Gradually, the microbubbles dissolve into the surrounded water, and the bubbles disappear with time [11–14].

Several forms of microbubbles with different characteristics have been studied in recent years [15–18]. The required characteristics of microbubbles can be classified as functionality, configuration, and biocompatibility. The functional characteristics are those that give it the ability to perform different tasks, including scattering performance by ultrasound (US), as well as the versatility to be injected [19–22]. Since these microbubbles should be delivered to the body in order to carry out their various operations, they should be injectable carriers [23]. This is the only factor for the use of microbubbles since they have the same efficiency as the US diffusion. It has been assumed that the US-mediated degradation of microbubbles may be a modern and revolutionary method for the minimally invasive delivery of drugs and genes to various tissues. Some tiny bubbles or microbubbles are intended to retain drugs or agents so that they can be inserted into a certain area of interest. But as these bubbles are destroyed for distribution, drugs or agents are released to various locations within the body [2, 24].

Methods that may be used for the preparation of these microbubbles include sonication, cross-linking polymerization, atomization, reconstitution, and evaporation of solvent emulsion [25, 26]. Sonication is preferred for the development of microbubbles involving US transmission or by entering the septic system with an ultrasonic probe requiring an ultrasonic vibrating hypodermic needle [27, 28]. The Sonication process can be applied to a variety of methods, such as a syringe consisting of a surfactant mixture and a gas at the top of the syringe that can be sonicated by a thin layer. Sonication can be achieved by depressing or contacting the layer using an ultrasonic or beam-based probe. Once the sonication phase has been completed, a combination of microbubbles can be collected from the syringe and delivered to the patient. Sonication also can often be achieved by using a low-power, ultrasonic vacuum assembly inside a syringe [29–31].

Microbubbles have been used in a variety of fields, including food and industry [32, 33], water purification, wastewater treatment and purification [34, 35], groundwater treatment [36], agriculture [37, 38], biomass processing [39], US sensor [40], hygiene [41], etc. The use of microbubbles in the field of medicine is now very promising and brilliant. Microbubbles or small spherical-shaped gas bubbles consist of phospholipids or environmentally friendly polymers are very small in size to approximately the size of red blood cells and are used in several forms in the biomedical field, including diagnostic loads, as drug carriers, and as a gene transporter in conjunction with the US [42–46]. Microbubbles have many fruitful uses in screening/diagnostic equipment [47–49], ophthalmology [50, 51], dentistry [52, 53], surgery [54], pharmacy [55], cardiovascular disease [56], inner ear drug delivery [57], brain delivery [58], renal diseases [59], cancer [60, 61] and so on.

In this paper, we highlighted microbubbles and their beneficial medicinal applications in the treatment of a number of prevalent diseases, including Parkinson's disease, Alzheimer's disease, cardiovascular disease, diabetic condition, renal disorders, and ultimately, their use in the treatment of different types of cancer as well as their application on the basis of the combination of US and nanoparticles.

## Microbubbles properties

### Microbubbles components

**Fig. 1:**
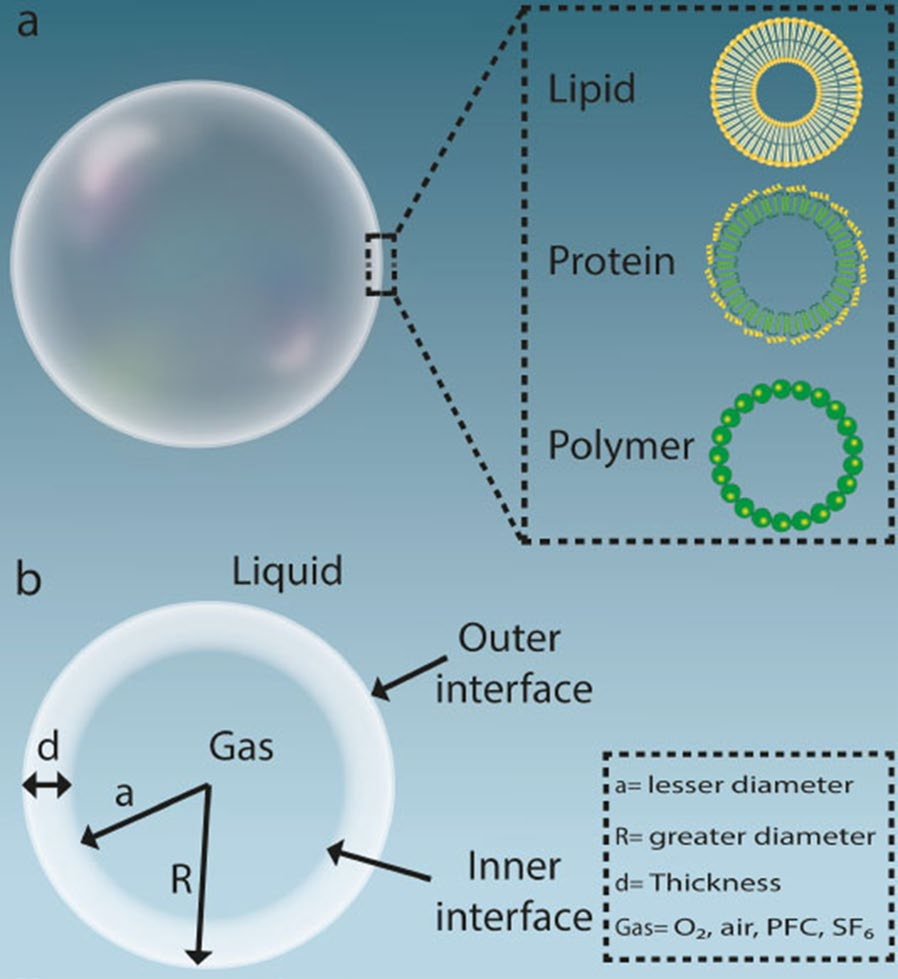
**a** Schematic illustration of a common microbubble. **b** Microbubbles components; *SF6* sulfur hexafluoride, *PFC* perfluorocarbon

Microbubbles consist mainly of three distinct phases: the internal gas phase, the shell layer substance enveloping the gas phase, and the external aqueous or liquid phase (Fig. 1) [62–64].

A single gas or a mixture of gasses may be used in the internal gas phase. A mixture of different gasses is used to create low-pressure gradients and to increase the pressure that sustains the bubble. When a mixture of gasses is encountered, two kinds of gasses are produced, one of which becomes the main altering gas, commonly considered to be the first gas. Air is assumed to be the predominant gas modifier. Nitrogen has also the potential to be the first gas modifier. The evaporation pressure of the first gas appears to be 760-x mm hg, where x is the evaporation pressure of the second gas. The second gas in this mixture is the osmotic part, which is more widely referred to as secondary gas. It is the ideal gas that is less penetrable on the surface of the bubble particles, unlike the alternating gas. It is therefore desirable since the gas osmotic substance is less dissolvable throughout the bloodstream and serum. The gaseous osmotic agent normally consists of a gas or liquid at room temperature so that it has sufficient vapor or partial pressure at the temperature of action to produce an adequate osmotic effect. Sulfur hexafluoride or fluorocarbon are two examples of secondary gas [65–68].

The key factor in the efficacy of microbubbles, mostly as a drug delivery carrier, is their potent behavior once subjected to ultrasonic wave radiation. The gas center persists during the rarefaction of the wave voltage and can be contracted during the compaction process. Based on US wave specifications, there may be different manifestations that promote US backscatter, delivery, and location of drug release from the microbubble layer. Such occurrences can have minor effects, such as acoustic radiation, or they can have highly reactive effects, such as inertial cavitation. The combination of these processes leads to better image analysis, controlled release of drugs, and improved vasculature permeability [69–71].

The gas phase is surrounded by a layer of the shell. It plays a significant role in the mechanical properties of the microbubble, as well as in the dispersion of gas from microbubble particles [72–74]. This layer also acts as a protective layer for the encapsulation of therapeutic agents. The ligands may be attached to the membrane layer to target these microbubbles to various other tissues or organs. This layer is responsible for the elastic modulus or the compressive strength of the microbubbles [75–78]. The more flexible the component of the shell layer, the higher the acoustic intensity. The shell layer may tolerate acoustic intensity until it breaks or bursts, which may increase the retention time of the bubble particles throughout the body [79, 80]. The more hydrophilic properties of the layer material, the easier it is to absorb the particles through the body, reducing the period of residence of the bubbles in the body. Various types of shell-forming substances can be used, such as proteins (e.g. albumin), phospholipids (e.g. phosphatidylethanolamine, phosphatidylcholine), biodegradable polymeric materials (e.g. polycaprolactone and polyvinyl alcohol), surfactant shells, and multi-layer polyelectrolyte shell layers [23, 81–83].

### The size of the microbubbles

Increased tissue permeabilization has been connected to the diameter of microbubbles. Samiotaki et al. used 2-, 4-, and 6-µm diameter microbubbles in mouse models to investigate the influence of microbubble diameter on a widely utilized BBB disruption metric, the measurement of the MRI contrast agent that is being extravasated [84, 85]. Although the study's primary goal was to determine the velocity during which the BBB healed following BBB disruption, multiple secondary findings presented compelling evidence in favor of using monodisperse microbubbles for enhancing treatment management. The results showed that the BBB recovery process happened in the reverse of permeabilization, beginning at the treatment area's outer edges and starting to move towards the central core, utilizing a 1.5-MHz focused US array for 60 s between the thalamus and the hippocampus on the right hemisphere, employing a 60 µs pulse length, 10 Hz pulse repetition frequency (PRF), and 0.3–0.6 megapascal at peak negative pressures [85]. The duration of BBB disruption was extended to almost five days using focused-US with bigger microbubbles (4 and 6-µm). The time it took for the BBB to close was also affected by the diameter of the microbubble.

With polydisperse commercial products, previous research showed that the duration of BBB closure was around 24–48 h. This result, according to the authors, is due to bigger openings formed by bigger microbubbles. Furthermore, tissue injury (dark neurons, extravasation of red blood cells from blood capillary) initially observed in short period investigations was not found in the mice, or was considerably reduced, demonstrating that disturbed tissue may heal following a few days. Nevertheless, because this investigation assessed scale effects at a typical microbubble number dosage (in microbubble/kg units) instead of a microbubble volume dosage, it is challenging to draw a distinction between the comparative impact of number versus dimension/size. As a result, it is unknown if the diameter of microbubbles impacts the level of BBB disruption when separated from the influences of microbubble number dose. Song et al.'s preliminary findings on microbubbles coated by cationic lipid show that it does not, but additional research is needed to validate this [86].

### Mechanism of action

The acoustic sensitivity of microbubbles with diameters of 0.1–10 µm, which are gas spheres encapsulated by protein or lipid, is crucial to microbubble-assisted focused-US [87, 88]. Because of their extreme compressibility and inclination to cavitate under US waves, microbubbles possess the ability to transfer kinetic energy from the traveling acoustic wave to the surrounding microenvironment [89]. Microbubbles fluctuate volumetrically while cavitating, causing fluid to flow within a size ranging far from their surface [90, 91]. As a result, the oscillation causes transient permeabilization and mechanofluidic impingement of neighboring tissues and cells.

Stable and transient acoustic cavitation are the most common types of cavitation. Bubble instability characterizes transient cavitation, which is frequently followed by severe inertial forces like fragmentation, jetting, and shock-wave generation [92, 93]. The latter causes a wideband acoustic emission, which is commonly referred to as "inertial cavitation". Because of the greater forces and pressures required to cause inertial cavitation and the harmful impacts found on inorganic substances, it has been proposed as a marker of cellular injury [94, 95]. During microbubble-assisted focused-US blood–brain barrier (BBB) disruption, these inertial occurrences are likely to cause vascular rupture and invagination [93, 96]. Nevertheless, the definition "inertial cavitation" is a misnomer since "stable" cavitation (characterized by persistent bubble movement) also can result in inertial effects with a high-Reynolds-number. Harmonic emissions are used to identify stable cavitation acoustically [97, 98].

In comparison to inertial cavitation, which has a shorter duration, stable cavitation has an identical, if not stronger, influence on cells because the cavitation strength lasts for a significantly longer period of time, transferring more net kinetic energy output. The presence of BBB disruption during steady cavitation has been established using passive cavitation detectors (PCDs) [99, 100]. It was discovered that switching from stable to inertial cavitation results in higher molecular weight transportation. Furthermore, microbubble-assisted focused-US alterations in the gene expression of essential BBB efflux transport proteins, like downregulation of the P-glycoprotein, may drastically affect the pharmacokinetics of BBB disruption, enhancing medication localization in the parenchyma [101, 102]. Nevertheless, no study has been able to separate the specific physical basis of stable cavitation on the BBB disruption due to the difficulty of precisely in vivo behavior of the detecting microbubble at the BBB at tissue depth.

### Pharmacokinetic behavior of microbubbles

After infusion or injection, microbubbles are capable of circulating throughout the body until the US stimulates the targeted location [103, 104]. The circulation durability of microbubbles is thought to rely on their dissolution rate and removal speed by the mononuclear phagocyte system. Individual microbubble parameters, including diameter and gas/shell content, and also the ensemble, impact the dissolution rate of microbubbles (size distribution and concentration) [90, 105]. For instance, evidence from in vivo imaging investigations and well-established models of individual microbubble dissolution implies that expanding microbubble dimension and/or increasing their concentration greatly enhances circulatory stability [4, 106].

The half-life of the microbubble and microbubble volume dosage were shown to have a linear connection in terms of disrupting the BBB. In addition to offering longer imaging windows, improved circulatory durability is capable of improving microbubble concentration at the targeted location during treatment, thus enhancing its permeabilization. O'Reilly et al. found that disrupting the BBB was directly linked to peak circulating concentration of the microbubble: bolus injections in short-duration (15 s) continuously influenced higher BBB disruption than long-duration (2 min) infusions of the identical microbubble dosing regimen, most probably owing to a decreased peak concentration of microbubble throughout the circulatory system for the latter. More research is needed to determine the function of microbubble and drug pharmacokinetics in microbubble-assisted focused-US BBB disruption, which could be facilitated through the utilization of microbubble volume dosage when administering [107].

## Novel microbubble-based treatment in biomedicine

**Fig. 2:**
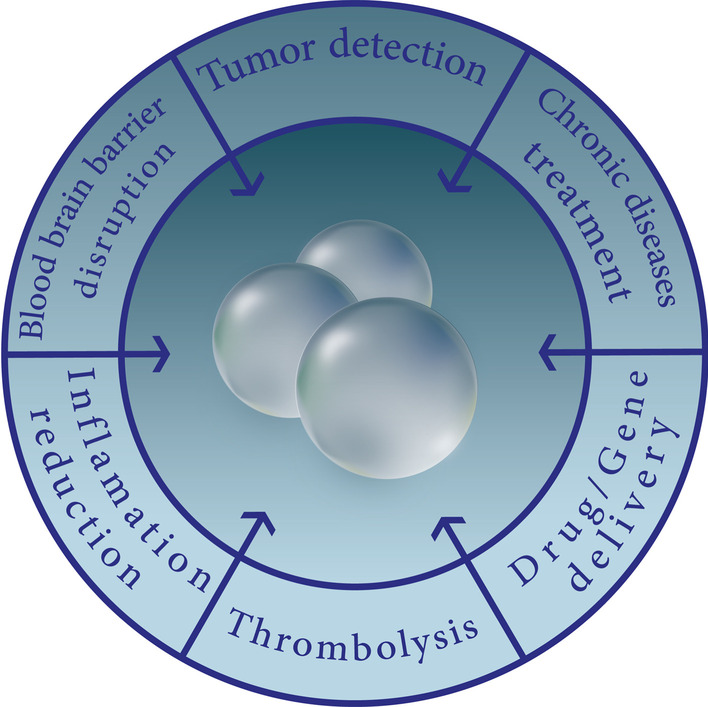
Microbubbles in biomedical applications

The unique ability of microbubbles to respond to the US makes them useful agents for the treatment of brain, cardiovascular, renal diseases, diabetes, and cancer (Fig. 2) [108].

### Brain disease treatment

#### Parkinson's disease treatment

**Fig. 3:**
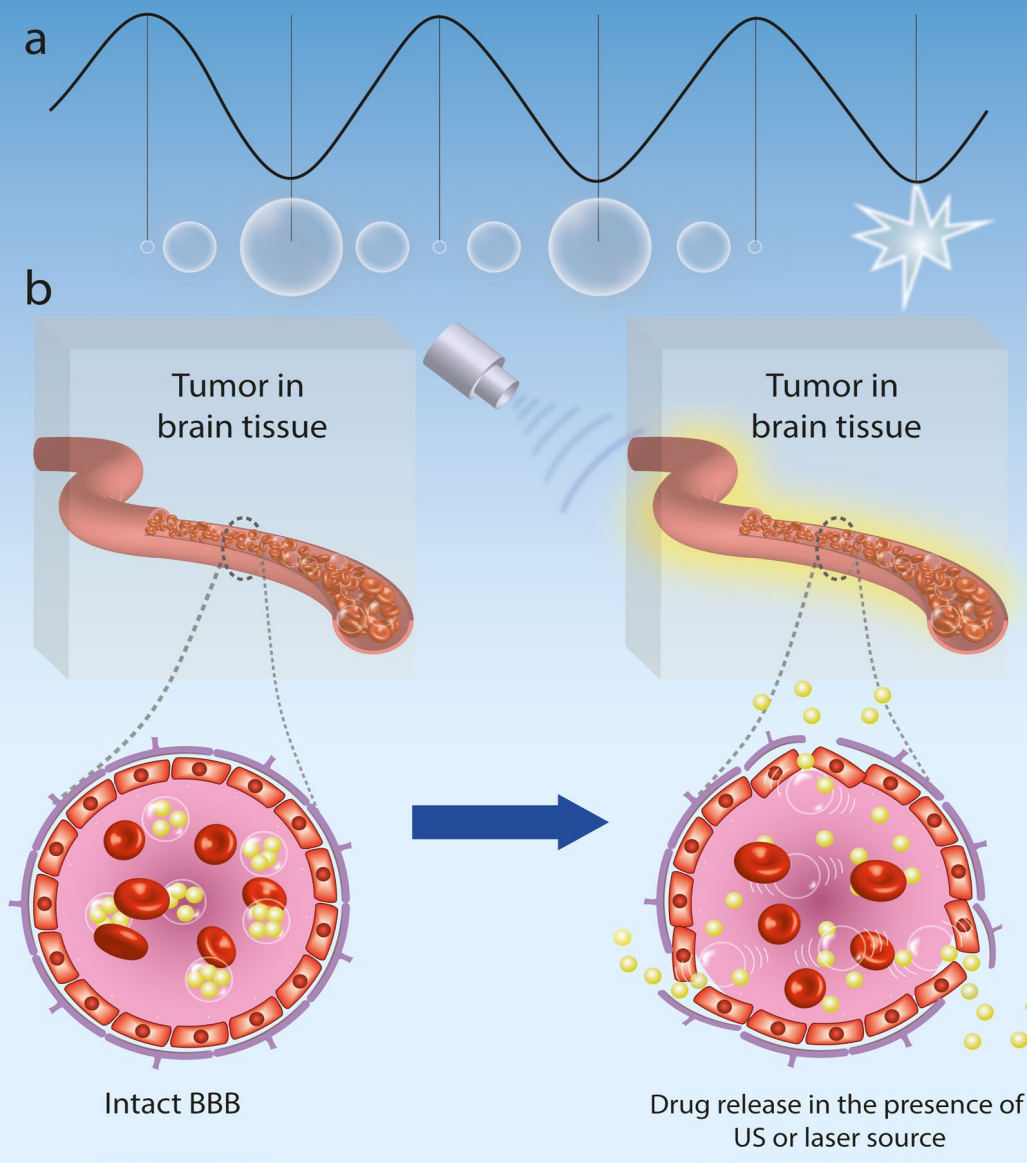
**a** Physical processes affecting the physiological activities mediated by activated US-induced microbubbles; **b** Schematic illustration of the US-induced microbubbles mechanism for opening the BBB

Acoustic cavitation is a physical process that emerges from the inside of the gas-filled bubble particles in the environment that have been introduced to trigger both harmonic microbubble expansion and regulated US intensity compression [75, 109]. US-induced microbubbles can physically interact with the surrounding environment through stable or inertial cavitation (Fig. 3a) [110]. Acoustic cavitation can also be distinguished by two physical processes that lead to the opening of the BBB: inertial cavitation and stable cavitation.

Stable cavitation leads specifically to a tight junctional disturbance, while inertial cavitation can induce excessive extravasation of erythrocytes from the bloodstream [111]. US stimulus tends to cause persistent microbubble oscillation in stable cavitation. The increased microbubble expedition distances the endothelial cells from each other, while the compression causes invagination throughout the vascular sheath, which can trigger the expansion of tight junctions through force-pull processes. Quick oscillation of microbubbles can also cause constant micro-streaming pressure between circulating microbubbles to induce adjacent vascular endothelium. This streaming theoretically adds shear stress to the cells, disrupts the endothelial layer's cohesion, and increases internal cell permeation. In addition, microbubbles can force the endothelium through the US, producing acoustic radiation as well as increasing vascular penetration [112] (Fig. 3b).

The BBB continues to remain one of the most severe impediments to gene therapeutic interventions for the central nervous system. Therapeutic genes are promptly picked up by the reticuloendothelial system and destroyed by nucleases throughout the bloodstream after they are introduced into the systemic circulation. In terms of disease-modifying techniques, researchers recently exhibited that an innovative delivery method, which combines gene-carrying liposomes with microbubbles, can transport the glial cell line-derived neurotrophic factor (GDNF) gene specifically into the 1-methyl-4-phenyl-1,2,3,6-tetrahydropyridine (MPTP)-triggered Parkinson's disease animal brain [113, 114].

Following intravenous injection of microbubbles, US energy generates microbubble oscillation, which intensifies the regional acoustic cavitation action that allows microbubbles to physically interact/interface with nearby vascular tissues through shear stress and mechanical microstreaming [75, 103]. When microbubble fluctuations emerge around endothelial cells, shear stress increases vascular permeability by temporal disturbance of endothelial junctions (for example, targeted BBB opening) or increased cell transcytosis activity [115, 116] As a result, Us-targeted microbubble destruction can improve the entry of larger therapeutic molecules into diseased central nervous system areas, thus improving gene-carrier uptake in damaged brain areas without destroying normal cells. The greater degree of sophistication of the conventional load-carrying approach and incubation conditions is another barrier to deliver therapeutic genes into the brain.

Quicker and easier techniques for manufacturing more generalized gene transporters should be developed to clinically operationalize the ultrasonic gene delivery paradigm. For example, the GDNF and neurotrophic brain-derived factor (BDNF) genes are the two most widely studied therapeutic genes in Parkinson's disease gene therapy, however, because of the complexities of the gene carrier architecture, only a few investigations have provided head-to-head assessments of their therapeutic effectiveness in Parkinson's disease treatment applications. Furthermore, only a few studies have looked into the possibility of simultaneously delivering BDNF and GDNF in Parkinson's disease gene therapy [117].

Parkinson's disease is characterized by differential destruction of dopaminergic neurons and by neural inflammation of the substantia nigra. There is, in fact, no definitive cure for delaying its development [118, 119]. Initial Parkinson's disorder treatment focuses on the administration and use of levodopa (L-dopa) that can infiltrate BBB and restore striatum levels of dopamine [120]. Even so, L-dopa therapy does not inhibit the progressive degradation of the dopamine-generating neurons involved in motor fluctuations and dyskinesia [121].

Microbubble destruction based on US-targeted neurotrophic factors in combination with the gene delivery system may promote infiltration of therapeutic genes across the brain for neuroprotective treatment of neurodegenerative disorders. US-induced microbubbles have been demonstrated to open BBB non-invasively, locally, and reversibly to energetic ranges that do not trigger cell destruction and damage that offer the potential to effectively manage brain disorders such as Parkinson's disease [101]. A novel gene delivery mechanism has been designed to incorporate liposomal microbubbles that carry BBB genes and enable microbubbles to release genes to the brain. Given that both GDNF and BDNF have been shown to protect dopaminergic neurons from the neural toxicity observed in Parkinson's disease experimental results, the latter study aims to develop a novel gene-nanocarrier microbubble structure capable of transporting the GDNF or BDNF gene and preserving dopaminergic neurons in Parkinson's disease. This microbubble-based gene delivery system exhibits neuroprotective properties, as evidenced by significantly reduced cognitive decline, decreased calcium inflow, decreased caspase3 and GFAP expression, and averted dopaminergic neuronal damage [117].

In another similar research, real-time MRI-guided US-based microbubbles containing a GDNF-borne retrovirus have been used to irradiate the substantia nigra of the animal model to open the BBB, allow the retrovirus to pass the BBB, deliver dopaminergic neurons at this location, and enhance the expression levels of GDNF within these neurons [122]. An innovative fluorescent probe has been developed for the bio-imaging of activated microglia that targets tiny molecules of FPR2/ALX via US microbubbles. This device did not co-localize with neurons or astrocytes but was collected in active microglia, allowing for image processing in future drug development projects focusing on neurodegenerative diseases such as Parkinson's disease [123].

An excellent microbubble-based gene delivery system has been developed that is triggered and activated by US-focused liposomes as therapeutic gene carriers. These microbubble-based carriers could actively deliver therapeutic genes to the brain and effectively induce GDNF and reporter gene expression. These complexes are capable of acting synergistically as an essential technique for the treatment of animals treated with MPTP and may have the capacity for enhanced gene therapy against neurodegenerative disorders (such as Parkinson's disorder) [114]. Liposomes modified with polyethylene glycol (PEG) and containing the GDNF plasmid gene conjugated with microbubbles are highly effective in treating behavioral abnormalities and neuron dysfunction associated with Parkinson's disease [124]. In another study, the ability of US-focused anti-alpha synuclein (a-syn) monoclonal antibody to deliver Parkinson's disease in combination with microbubble particles was investigated. The study's findings indicated that regular US-based anti-sync interventions could effectively reduce the burden of a-sync in Parkinson's disease[125].

#### Alzheimer's disease treatment

Neurogenesis in adults is a step that involves the formation, advancement, and integration of new nerve cells into the brain [126]. Neurogenesis in the dorsal hippocampus occurs through the dentate gyrus, which corresponds to learning and memory skills and may be deficient in neurological disorders such as Alzheimer's disease [127, 128]. Transcranial-based US-focused in combination with a microbubble contrast agent, which was subjected to a temporary increase in BBB permeability as an identified factor, was evaluated for its ability to trigger hippocampal neurogenesis. Focused US-based therapy in adult mice dramatically improved the frequency of propagating cells in the hippocampus dental gyrus. This supports the theory that US-based microbubble therapy can induce the process of neurogenesis in the hippocampal, a mechanism that encompasses learning skills and memory capabilities that are impaired by neurological disorders such as Alzheimer's disease [129].

During the care and management of diseases, the barriers to the central nervous system as a protective layer are challenging. In particular, the BBB is a major challenge for the delivery of contrast agents for image processing and also for the delivery of therapeutic substances to the brain [130]. The US-based gas microbubble was designed to temporarily open the BBB and effectively deliver silica-coated gold nanorods. This unique nanoagent showed a high optical absorption, enabling ex vivo and in vivo visualization of the prescribed particles via US-focused photoacoustic visualization. This experiment established the capability of longitudinal medical diagnostics using US-guided photoacoustic imaging of microbubbles. It was also used to monitor and track the therapeutic effects of microbubbles on neurological dysfunction [131].

Intelligent nano and microsystems, including microbubbles, have been developed to facilitate drug transmission across the BBB. In a novel survey, microbubbles were employed in conjunction with the US to open the BBB for the transportation and delivery of therapeutic agents. This system was created by enclosing a Quercetin-functionalized sulfur nanostructure in microbubbles. It could be destroyed instantly when exposed to ultrasonic waves and could improve the vascular system's penetrability, resulting in a floating expansion of the BBB due to the "sonoporation" response [132]. Quercetin-functionalized sulfur nanostructures were released from the outer shell of the microbubbles and penetrated the brain through the BBB opening, accumulating throughout the brain parenchyma. Due to the rapid absorption of these nanostructures throughout the brain, neuronal programmed cell death, calcium homeostasis imbalance, oxidative stress, and inflammatory reactions all facilitated by endoplasmic reticulum stress and preserved neural cells were significantly reduced, thus continuing to treat Alzheimer's disease efficiently. The evaluation results showed that the learning capacity and memory function of Alzheimer's disease in controlled mice with these nanostructures was significantly increased and no noticeable adverse effects were observed. This equipped microbubble in conjunction with the US provides effective and secure treatment for neurodegenerative disorders, as well as a potential method for endoplasmic reticulum stress therapy [133].

Zhu et al. used a dual delivery strategy on the basis of US-assisted microbubbles destruction for transportation of β-amyloid antibody loaded by microbubbles and neural stem cells on Alzheimer’s disease. It has the potential to be a successful dual delivery approach since it can successfully and safely improve BDNF expression, reduce beta-amyloid protein accumulation, and restore impaired learning and spatial memory capability as compared to a single Aβ antibody administration route [134].

The delivery of drugs to the brain is complicated and necessary to mitigate systemic toxic effects due to the presence of BBB and decreased delivery performance. Intranasal delivery of gold nanoclusters has been demonstrated in some studies to be more effective than systemic injection in minimizing overall toxicity [135, 136]. Relative to intranasal drug delivery to the brain alone, intranasal drug delivery based on US microbubbles resulted in a targeted and increased release of gold nanoclusters. The short-term safety assessment of the treated mice did not detect the destruction of the trigeminal nerve, nose, and brain tissue. These results indicate that intranasal drug delivery based on US microbubbles is an advantageous strategy for the spatially targeted, non-destructive, and safe delivery of nanostructures to the brain with reduced systemic toxicity and exposure [135].

Nanostructured poly(ethylene glycol)-poly(lactic acid) (PEG-PLA) material in combination with microbubbles modified by specific antibodies has been developed for the delivery of amyloid-beta peptide. Microbubble-enhanced non-focused US technique has also been used to promote the release of modified PEG–PLA nanomaterials or the identification of Alzheimer's disorder biomarkers that are widely distributed across the brain. Microbubble can dramatically improve the brain delivery of nanomaterials so that this strategy can contribute as a secure and scalable strategy for the future use of nanomaterials in the treatment and diagnosis of brain disorders [137].

### Cardiovascular disease treatment

With rapid economic growth, lifestyle changes, and urbanization, the proportion of people with cardiovascular disease is increasing worldwide. Although recent improvements have been made in the management and treatment of cardiovascular disorders, the main cause of death continues in so many countries. There is therefore a strong incentive to maintain effective treatment and prevention [138–140]. Thrombosis is usually the cause of myocardial infarction and ischaemic stroke, although they may also be considered the leading cause of death. Accelerated circulation of blood is needed to help improve heart attack and the consequences of stroke [132].

**Fig. 4:**
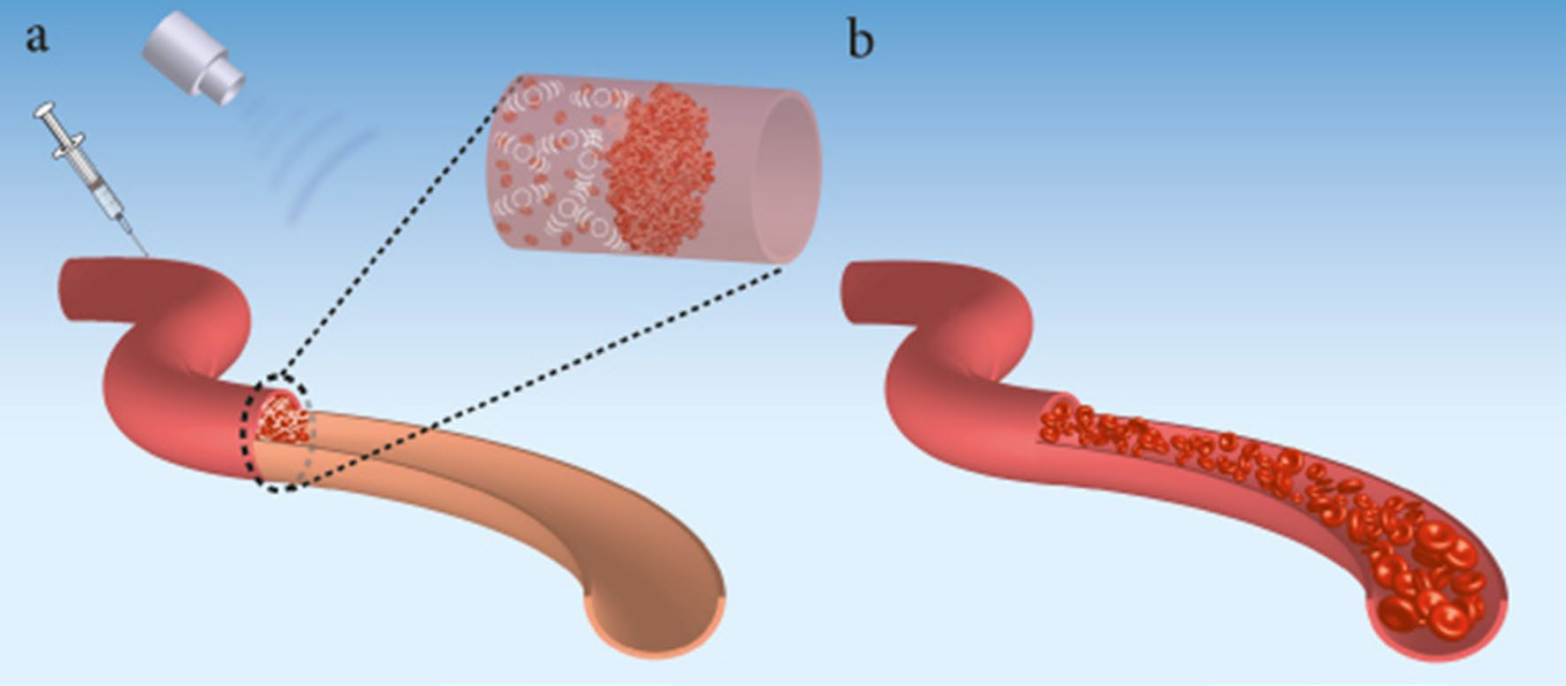
Improved sonothrombolysis of obstructive blood clots mediated with microbubbles. **a** Shows a blood vessel obstruction caused by fibrin clots, platelets, and RBCs. Microbubbles are injected (e.g. by intravenous infusion) and collected near the clot. **b** The US is used and cavitates the microbubbles that dissolve the blood clot and restore the flow. The mechanical bioeffects of ultrasonic-based microbubbles are used to enhance thrombolytic medications diffuse into blood clots and/or mechanically break down them. The commonly recognized thrombolysis progression concept is that the US-based microbubbles can generate inertial cavitation, micro-streaming, acoustic radiation, and steady cavitation force to temporally “loosen” clots of fibrin and promote thrombolytic medication distribution/diffusion, leading to faster and more precise clot treatment. Also, using the US-based microbubbles strategy, the degree of force generated and, as a consequence, the degree of clot displacement is controlled by adjusting the transducer's acoustic intensity and center frequency

The employment of the US to improve thrombolysis therapy is known as sonothrombolysis [141, 142]. Figure 4 shows sonothrombolysis during severe vascular occlusion caused by blood clots. Since the late 1980s, physicians have been using US-enhanced thrombolysis. Sonothrombolysis is frequently used to aid in the improvement of thrombolytic therapy results [143, 144]. This therapeutic method uses ultrasound's mechanical bioeffects to assist thrombolytic medicines to diffuse into blood clots and mechanically break down blood clots [89, 145].

The frequently accepted principle of thrombolysis advancement is that the US can cause inertial cavitation, micro-streaming, acoustic radiation, and stable cavitation force to temporarily “loosen “ clots of fibrin as well as enhance thrombolytic diffusion of drugs, enabling for quicker and more accurate clot treatment [146–149]. Several investigations and clinical studies have shown that ultrasonic contrast agents, specifically microbubbles, can be utilized to improve clot lysis with tissue plasminogen activator (tPA) by enhancing the quantity of directed cavitation, therefore eliminating blood clots while reducing clot debris (Fig. 4) [147, 150–158].

Triggering clot displacement is one well-established strategy for improving thrombolysis [146, 147, 150, 159–163]. The acoustic radiation force is one of the most important acoustic processes for clot displacement [150, 156, 161, 163, 164]. The acoustic amplitude, frequency-dependent attenuation, and sound speed of the medium are all factors in the calculation of acoustic radiation force [165]. From this, we can see how adjusting the transducer's acoustic strength and center frequency affects the amount of force created and, as a result, the amount of clot displacement. As a basis, the acoustic characteristics and transducer construction employed should be considered concerning the processes of sonothrombolysis. Cavitation is also another generally acknowledged process for sonothrombolysis [150–152, 159, 166, 167].

Inertial and stable cavitation are both included [168, 169]. Displacement and cavitation contribute to acoustic streaming, fibrin disaggregation, thrombolytic penetration, as well as breaking up clots. Cavitation is assumed to be a process that occurs both without and with additional contrast agents. Cavitation can be created without contrast agents in high-intensity focused US and histotripsy, although using microbubble contrast agents lowers the threshold of the cavitation. Cavitation may be caused by cells inside blood clots acting as nuclei, resulting in further clot breakdown [170].

Enhanced thrombolytic results are also assisted by acoustic streaming [171–176]. Acoustic streaming is associated with the improvement of thrombolytic and US contrast agents penetrating into clots by stimulating diffusion, carrying thrombolytic substances, plus mechanical disturbance [158, 177, 178]. Another theory is that ultrasound causes a redistribution of fibrin and thrombin on the clot surface, permitting stronger penetration within the clot. It has also been suggested that sonothrombolysis promotes fibrin disaggregation [177, 179]. Biological factors, including the existing thrombin limitation that is depleted from existing plasma, activation of an enzyme, and US-activated platelet, are also hypothesized as mechanisms [174, 180–184].

While it was anticipated that temperature might impact enzyme activity and promote diffusion, the majority of investigations have revealed no significant link between thermal processes and thrombolytic results [147, 156, 185–191]. Also, sonothrombolysis relies on the cavitation of microbubbles, which is a key process. There is an impact of US without microbubbles when ultrasonic intensities are within the range approved by the FDA for medical imaging, but the influence is increased when microbubbles are involved [24, 103, 192, 193]. High-energy US pulsing is typically punctuated by timeframes without high-energy pulsing in sonothrombolysis; during these durations, reduced mechanical index US imaging could be used to supervise and track the increase in the number of fresh microbubbles in the thrombosis area (Fig. 4). Fresh microbubbles can enter the ultrasonic field during the durations when there is no high-energy pulsing [157].

An abdominal aortic aneurysm (AAA) lesion is a permanent and irreversible convex in the arteries with a higher incidence in the elderly. Increased aneurysm size by time is a lethal event that can contribute to the rupture of the sidewall. Aggressive surgical procedures are essential to prevent the production of AAA. Even so, such techniques have seriously harmful consequences. Targeted delivery of drugs using microbubbles has been commonly used to inhibit the development of AAA. Microbubbles with a dimension of 4.5 μm perform better function than other microbubbles in the targeted drug delivery mechanism to the internal wall of the AAA in a non-invasively route [194].

By incorporating cationic lipids into the stabilizing structural component, RNA and DNA may be electrostatically attached to cationic microbubbles. According to research findings, when more than 10% of the shell lipid is replaced with the cationic lipid, the microbubbles may become unsustainable. By adding cationic polymers (e.g. polyethyleneimine) to the surface of microbubbles, the researches have shown that the entrapment efficiency of microbubbles for genetic materials [195–197]. The US-targeted microbubble destruction may be able to deliver higher volumes of genetic material or gene-based medicines using this method.

In this regard, Microbubble destruction caused by the US and the corresponding release of miRNA can increase the absorption of miRNA in cardiomyocytes and increase the efficacy of treatment. This innovative delivery approach has the capability to further advance as an active targeting approach for miRNA therapy. This drug delivery approach may be widely used for other organs and diseases that are susceptible to US delivery, such as solid tumors or the vascular system [198]. Metabolically, microbubbles are inactive, for example, they do not activate the immune response of the host [24].

Unlike bare DNA, which is inserted directly intravascularly, the genes attached to the microbubbles may be transferred to the tissue without being metabolized [199]. The DNA transported through microbubbles has been shown to be resistant and preserved by the degradation of US microbubbles following the release of DNA [69, 200]. New research has shown that, in conjunction with US degradation, microbubble-covered albumin particles can be used to efficiently deliver adenoviral transgene to myocardium of rats. Microbubbles containing β-galactosidase transgene attached to their layer have been injected into the jugular vein of the rat and dissolved through US mediation. Nuclear staining found that in the β-galactosidase-containing US-triggered microbubble, the hearts of mice showed a tenfold increase in production of β-galactosidase compared to control populations. Intriguingly, in the case of microbubble degradation with transgenic injection in one of the five control populations, the function of β-galactosidase was twice as high as in the other control groups, suggesting that membrane disturbance is a critical component of viral transmission [201]. Similar findings were made using US-based microbubbles consisting of CMV-luciferase plasmids. Luciferase function has been shown in rat hearts following the US, with insignificant expression among other organ systems [202].

Microbubbles of perfluorocarbon-exposed sonicated dextrose albumin (PESDA) have been shown to facilitate the release of engineered antisense oligodeoxynucleotides into the carotid artery, an antisensic nucleotide that inhibits restenosis. Accumulation of oligodeoxynucleotides across the carotid vascular wall was observed to be dramatically increased when administered to PESDA-bubbles and when subcutaneous US was used in the carotid artery, resulting in a dramatically lower risk of restenosis [203]. There was also a relatively low level of plasmid chloramphenicol acetyltransferase (pCAT) in canine hearts after intramuscular infusion. Interestingly, this analysis used liposomes with positive charges that could bind to DNA with negative charges [204].

### Diabetes disease treatment

US-induced microbubble destruction is also expected to play a pivotal role in all aspects of diabetes therapy in the coming years. Several other systematic reviews shed light on the significant benefits of such an approach in patients with diabetes [205–208]. Two hundred thousand people are affected by diabetes, which is the sixth most commonly debilitating condition in the world. Diabetes is a severe endocrine condition characterized by persistent hyperglycemia, including fat, carbohydrate, and protein processing abnormalities, insulin production triggers, or insulin action [208, 209]. Diabetes has been categorized into three major subgroups: type 1 (insulin-dependent) diabetes, type 2 (non-insulin-dependent) diabetes, and type 3 (gestational Mellitus) diabetes [210]. Both type 1 and type 2 diabetes require either full or partial reconstruction of pancreatic β-cells, which can then be recovered by one of the medical procedures used to regenerate beta-cell islets. As the level of beta-cell alterations across the human pancreas appears to be slow, in particular the following injury, medical regeneration attempts to develop novel methods for either beta-cell neogenesis or replication [211].

In potent research, human islets were grafted into the liver using portal veins, and VEGF (human endothelial growth factor) was overexpressed throughout the liver by US-induced destruction of microbubbles, leading to increased neovascularization and improved activity of the transplanted islet. The latter provided a clear indicator to the population that islet cell transplantation may have been an effective medicine for the treatment of diabetes, in which US-induced microbubble destruction allows almost non-destructive gene delivery to pancreatic islands with an efficacy similar to the regulation of β-cellular functions in the elderly animals [212]. In terms of enhancing the functionality of β-cells, it has been shown that gene transmission within islet β-cells can be effectively achieved through the use of rat insulin promoter plasmid with US-induced microbubble destruction innovations. This mechanism could facilitate the effective expression of glucose genes in animals treated with insulin promoter-luciferase plasmid in rats. Besides, rat insulin promoter–human-insulin plasmid has been systematically administered to the islets, leading to an improvement in the bloodstream of human C-peptide and a reduction in blood sugar (glucose) levels.

Also, rat insulin promoter–hexokinase-I plasmid was administered, culminating in an increase in the expression of hexokinase I protein in the islets and the insulin in the bloodstream. The research also introduced an innovative procedure for localized gene expression aimed at β-cells by a functionalized insulin promoter with intravenous microbubbles in rats. In this study, ultrasonic waves in the pancreatic microvessels disrupted the delivered plasmid DNA [213]. Beta-cell regeneration is a very promising idea. Interestingly, this procedure provided for the reconstruction and recovery of in vivo β-cell volume islands, as well as for the normalization of blood glucose, C-peptide, and insulin in rats without viruses. In this experiment, the human ANGPTL8 gene, which facilitates the spread of pancreatic β-cells, was administered to rat pancreas via US-induced microbubble destruction, leading to an increase in β-cell volume, an increase in glucose tolerance, and also an improvement in fasting blood insulin levels. In particular, despite the use of viral vectors, microbubble destruction with US waves allows different transcriptional genes correlated with cell production and function—betacellulin as well as pancreatic duodenal homeobox-1, NeuroD1, Nkx2.2—to be directly transferred to dysfunctional pancreas using US energy, resulting in beta cell regeneration [214].

Recent research has suggested a creative strategy in which the non-viral gene can directly target pancreatic islets using in vivo US-induced microbubble destruction engineering. Treated experimental prototypes received a gene cocktail consisting of genes regulating the cell cycle and proliferation, resulting in a strong and long-term reconstruction with normalization of blood insulin, glucose, and C-peptide levels. Overexpression of genes can contribute to the regeneration of β-cells, which can be achieved by the proliferation of current β-cells or by progenitor cell differentiation since both genes are able to induce unmanageable cell proliferation and even tumor formation [215, 216].

Coenzyme Q10-charged liposomes for initial diabetic nephropathy therapy have been formulated and mixed with US microbubbles. The findings showed that the development of diabetic nephropathy could be reversed at an early stage [217]. Substantial in vivo data have confirmed that nanomaterials combined with UTMD demonstrate high translational capacity in the delivery of Acid Fibroblast Growth Factor (aFGF) to prevent diabetic cardiomyopathy. Furthermore, this one-of-a-kind drug delivery platform allows for the targeted delivery/release of diabetic hearts with a variety of other biochemical macromolecules with aFGF-related physiological functions. When the appropriate dosage of the medicine is defined and the delivery method is fully standardized, this approach, in addition to the most widely used glucose regulation drugs, will equip diabetic patients with an efficient and practical technique to prevent diabetic cardiomyopathy and also to avoid hazardous health problems [218].

### Renal disease treatment

Chronic renal disease has recently become a serious socio-economic and health problem [219]. Renal interstitial fibrosis is a typical final mechanism for the development of almost all forms of chronic renal disease to end-stage renal disease. The appropriate inactivation of renal fibrosis may therefore be a key approach to the prevention of chronic renal disease [220].

In a novel drug delivery mechanism, the polylactide-co-glycolide (PLGA) nanostructures loaded by the PPARδ agonist (rosiglitazone, RSG) (the PLNPs-RSG complex) were designed in combination with the charged microbubbles. This innovative structure demonstrated increased in vitro cell uptake and in vivo renal targeting performance. In the unilateral ureteral obstruction rat protocol, the combination of the PLNPs-RSG-MBs matrix and the US application significantly reduced collagen accumulation and effectively reduced the process of renal fibrosis, and can be a potential route to the treatment of renal interstitial fibrosis [221]. As a useful strategy, a short-haired RNA (shRNA) targeting the connective tissue growth factor (CTGF) was cloned in a plasmid and loaded onto the cationic microbubble surface. This research has shown that CTGF shRNA delivery by US-targeted microbubble is capable of inhibiting CTGF expression so that specific therapy can improve progressive renal fibrosis [222]. The availability of renal components (such as intracellular portion) to elevated molecular mass drugs can be regulated by US microbubbles [223].

## Potential microbubbles platforms for fighting against cancer

Given the apparent increase in cancer survival rates, some persistent types of the disease still place a considerable burden on the patient population and health care services [224]. Human survival and wellbeing are seriously endangered by malignant tumors [225, 226]. There have been several research papers in the last 10 years on the use of medication-loaded microbubbles with the US for medication delivery in animal tumor models (Table 1). The preclinical investigation of drug-loaded microbubbles, especially when combined with the US for tumor treatment, has recently gotten a lot of press. The treatment of liver cancer and brain tumors was the subject of several investigations. In addition, some papers focused on diseases such as breast cancer, pancreatic cancer, and others. Chemotherapy medications transported by microbubbles can enter the tumor region through the circulatory system following intravenous administration, unlike conventional chemotherapy methods. When ultrasonic irradiation causes microbubbles to rupture in tumor tissue, medications carried by the microbubbles are released, which should be beneficial in the treatment of malignant tumors. Traditional chemotherapy is mostly unsuccessful in such situations and causes harmful side effects. The targeted release of chemotherapy agents will further focus on improving both the tumor response and the patient's response [227]. This leads to the conclusion that there is an urgent need to develop appropriate strategies based on US-induced microbubbles as an individualized drug treatment. US-induced microbubbles can be used to address barriers to cancer treatment [228].

**Table 1:** The use of drug-loaded microbubbles in conjunction with the US to treat tumors

Medication	Tumor type	The diameter of the microbubble	Microbubble type	Application	Reference
DOX	Glioblastoma multiforme	1.04 μm	Lipid-based	Evaluating the efficacy of BBB opening and medication transportation	[229]
DOX	Malignant glioma	4.00 μm	Lipid-based	The suppression rate of human glioblastoma cells is being investigated	[230]
DOX	Pancreas carcinoma	1.02 μm	Lipid-based	Pancreatic cancer treatment using a rat model	[229]
DOX	Liver tumor	1.50 μm	Poly(lactic acid)-based	Treating liver tumors in a rabbit model	[231]
DOX	Breast cancer	1.64 μm	Lipid-based	Examining the anticancer activity in human breast cancer cells	[232]
Paclitaxel (PTX)	Ovarian cancer	1.80 μm	Lipid-based	Treatment of ovarian cancer in a mouse model	[233]
PTX	Ovarian cancer	1.80 μm	Lipid-based	Evaluating the anticancer activity for ovarian carcinoma cells in human	[234]
PTX	Breast cancer	1.68 μm	Lipid-based	Breast cancer treatment efficacy in a mice model	[235]
Docetaxel	Colon adenocarcinoma	3.30 μm	Lipid-based	Researching the anticancer function on human colon adenocarcinoma cell line	[236]
Hydroxycamptothecin	Liver tumor	1.48 μm	Lipid-based	Accelerating the inhibitory rate of tumor	[237]
Carmustine	Glioblastoma multiforme	1.32 μm	Lipid-based	Assessing the glioma therapeutic effectiveness in a rat model	[229]

### Breast cancer therapy

**Fig. 5:**
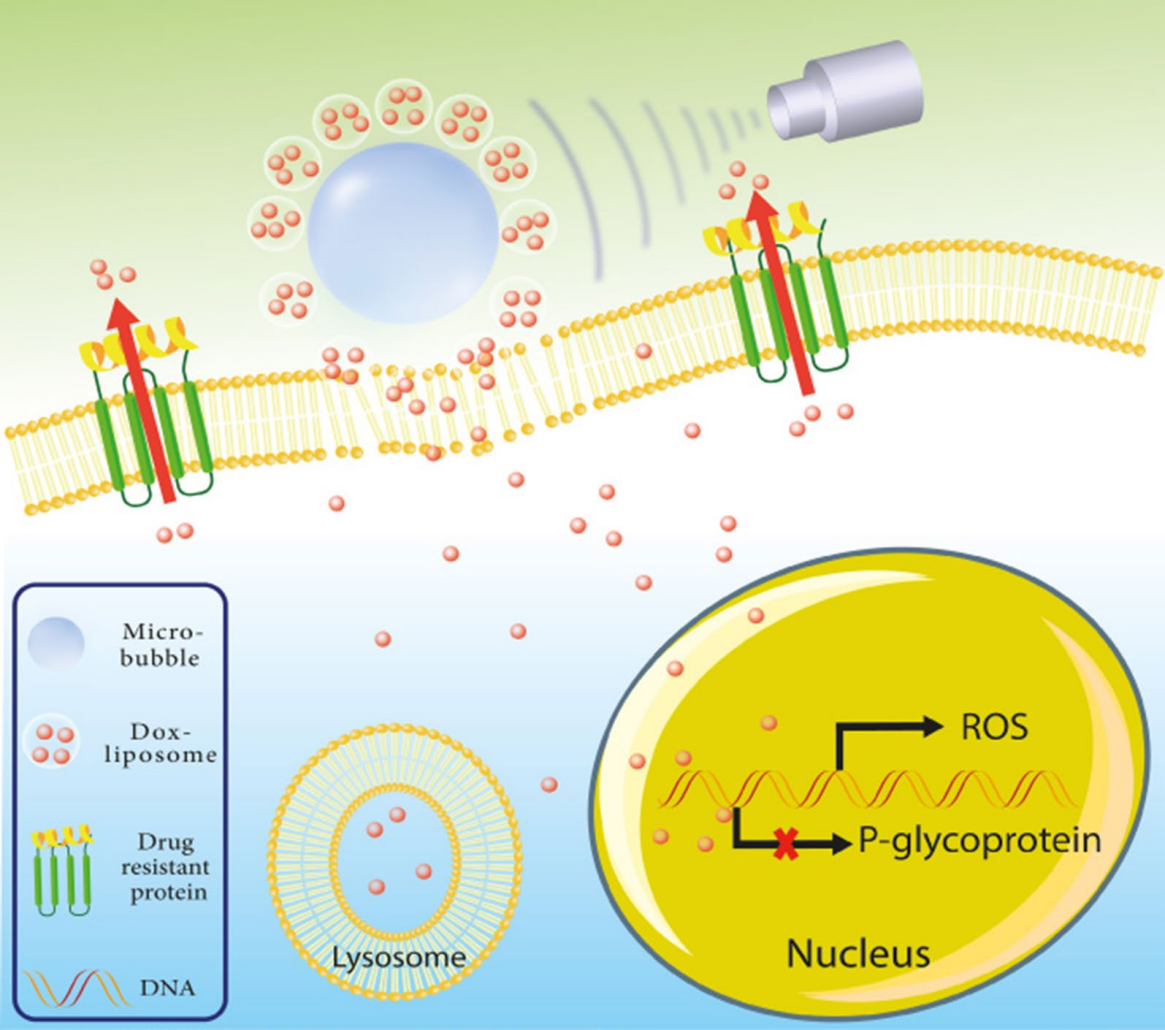
Graphic representation of doxorubicin-liposome-microbubble complexes (DLMCs). In the presence of the US, DLMCs have facilitated the reversal of multidrug-resistant phenotypes and counteracted them in human breast cancer cells

Breast cancer is one of the most prevalent types of cancer in women, and every year around 500,000 women around the world die from breast cancer. Data show that death rates and incidence of breast cancer have continued to rise; the median age at which women are diagnosed with breast cancer has recently become younger [238, 239]. PTX-loaded lipid microbubbles were provided using a mechanical vibration procedure. The LyP-1, a breast tumor homing peptide, was then coated to the surface of PTX-loaded lipid microbubbles by biotin-avidin grafting. Targeted ultrasonic microbubbles have achieved adequate drug-loading efficacy and have resulted in a high concentration of the formulated drug with excellent acoustic-induced destructibility, with a minimum half-life of approximately 43 s. The attachment of targeted microbubbles to human breast cancer cells has been extremely effective and durable, albeit with ultrasonic radiation exposure. Such creative therapeutic microbubbles are capable of releasing their cargo when exposed to the US. The efficiency of the cellular absorption of targeted microbubbles was higher than that of non-targeted microbubbles. More importantly, the anti-cancer effects of targeted PTX-loaded microbubbles along with the US have increased significantly. Research has shown that LyP-1 coated PTX-charged microbubbles have the potential to be used as a possible chemotherapy carrier for breast cancer therapy [240]. Also, the possible effects of the US-mediated destruction of PTX-loaded lipid microbubbles on MCF-7 apoptotic cell death suggest that the use of this complex along with the US can be an appropriate technique for breast cancer therapy [241]. Applying US ruptured oxygen-carrying microbubbles to radiotherapy tends to slow tumor development and improve the viability of a metastatic murine breast cancer model [242]. Subjecting human breast tumor xenograft model to US-triggered microbubbles increases tumor cell death and vascular damage due to hyperthermia. Appropriate therapeutic samples were determined to consist of 40 min of low-strength US therapy microbubble heating of 1 min of sonification and 1% of microbubble concentration accumulation [243]. The generation of localized microbubbles with intense diffusion in the tumor microenvironment is achieved by the construction of an integrated electrochemical simulation on a microstructured silicone needle coated with zinc-oxide nanowires. As a result, microbubbles are destroyed by external ultrasonic actuation-excitation, which causes micro-cavitation of cancer cells, followed by the subsequent penetration of anticancer agents in cancer cells. This platform, the ZnO nanowire-based microbubble generator probe, was studied in models of tumorized mice. Treated mice with this platform were shown to have an 82% decrease in tumor volume after 10 days with only 25% of the traditional dose of PTX, although only a 15% decrease in tumor volume was observed in the absence of the procedure. The presence of ZnO nanoparticles on microneedles significantly reduces the volume of microbubble and increases the efficiency of sonoporation [244]. An effective technique to overcome P-gp drug efflux pumps in resistance breast cancer cells has been demonstrated by targeted drug delivery to the nucleus using doxorubicin-liposome-microbubble complexes in combination with the US. A very rapid intracellular absorption of DOX was observed when the cells were treated with these complexes, and continuous nuclear accumulation of DOX was very frequently detected. Improved delivery of drugs and cell uptake has resulted in a significant increase in cytotoxic effects in MCF-7/ADR breast cancer cells (Fig. 5) [232].

### Lung cancer therapy

Lung cancer is indeed the type of cancer with the highest incidence and death rates in the world [232]. Anti-PD-L1 monoclonal antibody (mAb)-grafted and docetaxel-encapsulated multipurpose lipid-layered microbubbles have been designed with biosafe phospholipids to develop synergistic anti-tumor activity, mitigate adverse effects, and facilitate therapeutic benefits during US irradiation. These modified microbubbles had excellent cell absorption compared to free docetaxel, especially when combined with US exposure. It also resulted in increased levels of apoptotic cell death and increased levels of G2-M capture in cancer cells, which was strongly associated with PD-L1 expression. In vivo analysis found that synergistic therapy has excellent effects on tumor suppression, improved survival, and reduced negative effects. It also has an excellently controlled plan for immunotherapy and cancer treatment and a significant medical outlook for non-small cell lung cancer chemotherapy [245]. In another study, it was shown that overexpression of microRNA-449 inhibited the development of lung cancer. In addition, the US-microbubble-mediated miR-449a improved the repressive influence of miR-449a on the progression of lung cancer and may provide a unique perspective in the treatment of lung cancer [246]. Targeted microbubble based on US-destruction can be designed to facilitate the transmission of microbubbles loaded with EGF receptor (EGFR)-directed siRNA to murine squamous cell carcinomas. This platform can reduce the level of EGFR expression and EGF-associated development and increase the duration of tumor duplication [247].

### Glioma treatment

Brain tumors have a limited prevalence but an increased level of destructive power compared to many other tumors. Glioblastoma multiforme was the most common major brain tumor and is extremely malignant [248]. In the research provided, 1,3-bis(2-chloroethyl)-1-nitrosourea was effectively integrated into microbubble membranes, contributing to improved in vivo drug half-life and regulated release of drugs across brain tissue through targeted US sonication. Microbubble used in conjunction with US-focused is a hopeful method to carry chemotherapy drugs through BBB and increase specific drug accumulation in targeted brain locations, thereby reducing systemic cytotoxic activity [229]. MB-focused US BBB opening has been used as a promising novel multifunctional brain tumor drug delivery system [110].

The possibility of improving the response of PTX liposome to intracranial glioblastoma nude mice using prototype-focused US microbubbles has been investigated. The findings revealed that US focus combined with microbubble resulted in significantly higher BBB permeability. Quantitative and qualitative analyzes have shown that the absorption of liposomes produced by these non-intrusive approaches to glioblastoma has significantly improved relative to the level of infiltration of leaky blood vessels with cancer [152]. MRI experimental assessment found that intracranial glioblastoma development has been significantly inhibited by nude-treated mice on this platform. In the meantime, the viability of these nude mice has increased considerably. Immunohistochemistry research also verified anti-proliferation activity and cell apoptosis caused by improved delivery of PTX liposome through US-focused and microbubbles. Such results are intended to provide valuable knowledge for the application of minimally intrusive US-focused low-energy microbubble as an efficient method for delivering PTX liposomes and enhancing glioblastoma chemotherapeutic efficacy [152]. Additional sonodynamic therapy as a potential therapeutic approach to the delivery of sinoporphyrin sodium for intracranial glioblastoma therapy has also been developed [249].

### Liver cancer therapy

Liver cancer is currently commonly referred to as cancer, with an unfavorable prognosis and increased mortality in patients with liver cancer. Therapeutic options for liver cancer include radical surgical resection, local ablation, arterial chemoembolization, radiotherapy, and systemic chemotherapy. Even so, such clinical effects are still insufficient [250].

The new Glypican-3-targeted, curcumin-loaded microbubbles with increased drug loading capacity have excellent targeting capabilities for specific HepG2 cells used for sonodynamic therapy in liver cancer. Results have shown that sono-photodynamic therapy has been significantly more effective than conventional photodynamic therapy or sonodynamic therapy for the suppression of liver cancer. These microbubbles as sono/photosensitizers are stronger than curcumin-loaded microbubbles or curcumin alone. Sono-photodynamic treatment with Glypican-3-targeted, curcumin-charged microbubbles may be a promising route for the treatment of liver cancer [251]. Besides, a curcumin-loaded poly(l-lactide-co-glycolide) microbubble complex has been developed as a potential mechanism for the treatment of liver cancer [252].

Hepatocellular carcinoma is a cancer that is aggressive and has a bad prognosis because of its increased risk of metastasis [253]. Cancer stem cells have been implicated in tumor growth and are thought to be formed through epithelial-mesenchymal transition features [254]. CD133 is a surface hallmark specific to liver cancer stem cells, which also is a critical physiological determinant for carcinogenesis and total survival rate in hepatocellular carcinoma [255]. US-targeted microbubble destruction has been employed recently as a unique, secure, and successful method of gene transfection. Liu and colleagues investigated the regulatory mechanisms behind CD133 and the epithelial-mesenchymal transition in liver cancer stem cells, as well as whether US-targeted microbubble destruction based on the shRNA delivery approach enhanced gene trafficking in liver cancer stem cells [256]. CD133 positive cells were obtained from the SMMC-7721 liver cancer stem cell line and subsequently transfected with shCD133 using US-targeted microbubble destruction or liposomes. In comparison to the liposomes group, the US-targeted microbubble destruction group achieved much higher transfection efficiencies. CD133 silencing reverted the epithelial-mesenchymal transition process, inhibited proliferation, self-renewal, and migration of CD133 positive liver cancer stem cells, and inhibited the formation of cancer stem cell tumor xenografts. Furthermore, CD133 dysregulation resulted in a decrease in the nuclear factor-B (NF-B) pathway. CD133 is required for the control of the epithelial-mesenchymal transition mechanism, tumor-initiating characteristics, and migratory capacity of liver cancer stem cells, as established in their work. The US-targeted microbubble destruction approach may be investigated as a potential therapy option for hepatocellular carcinoma [256].

### Other tumors

Other cancer diseases, such as melanoma, ovarian cancer, metastatic colorectal cancer, and pancreatic cancer have been studied using the drug-loaded microbubble-based treatment in combination with the US, in addition to breast cancer, brain tumors, and liver cancer [257–260]. Tinkov et al. developed lipid-based microbubbles loaded by DOX and assessed their effectiveness in a mouse model bearing pancreatic cancer [261]. Ren et al. also used freeze-drying to create lipid-based microbubbles loaded by DOX, and their anticancer activity on a human colon cancer cell line was investigated [236]. The lyophilized microbubbles were kept in the form of freeze-dried powder, which was easier to carry and store. Yan and colleagues created PTX-loaded liposome-based microbubble complexes [235]. PTX liposomes were attached to the surface of microbubbles by avidin–biotin linkage in the mentioned work, which enhanced the drug-loading potential of microbubbles [235].

Concerned that drug-loaded microbubbles had shorter processing times and, as a result, bubble sizes were in the micrometer range, preventing the bubbles from crossing tumor tissue, Rapoport et al. suggested that microbubble prodrugs be developed initially, and afterward converted to microbubbles at the tumor location using the US, resulting in a therapeutic outcome [262]. Perfluoropentane nanoemulsion was prepared using the poly (ethylene oxide)-co-poly (l-lactide) copolymer, which could be transformed into microbubbles under ultrasonic irradiation circumstances or elevated temperatures. The anticancer activity of nanoemulsion coupled with the US was next demonstrated by transplanting human pancreatic cancer and ovarian cancer cells into mice. Nevertheless, following the primary stage of treatment, tumor relapse was detected, and it was determined that continuing therapy in similar regions was ineffective [262].

Before chemotherapy and radiotherapy, oxygen treatment is frequently utilized. It has the ability to induce tumor oxidation, increase medication absorption, and increase therapeutic efficacy. Microbubbles can be employed to provide oxygen and anticancer medicines at the same time [263]. Wang et al. created lipid-based microbubbles loaded with PTX and oxygen and evaluated their effectiveness in the treatment of ovarian cancer using microbubbles and the US [233, 234]. An in vitro study found that lipid-based microbubbles loaded with PTX and oxygen in conjunction with the US provided a synergistic impact on hypoxic ovarian cancer cells that are resistant to PTX [258]. It was discovered that using oxygen and lipid microbubbles loaded with PTX in combination with the US, it was possible to deliver oxygen and anticancer medications at the same time, resulting in greater anticancer effectiveness [258].

## A secure clinical setting for microbubbles

Microbubbles were initially used in a clinical setting in 1969 [264]. When employing B-mode sonography, microbubbles inside the blood pool are implemented to increase the signal-to-noise ratio or contrast of blood [265]. As a result, ultrasonic contrast agents are also described as microbubbles. Their diameters vary from 2 to 5 µm, with approximately 95 percent being less than 10 µm, allowing the microbubbles to move across the lung capillaries [266]. Microbubbles, like the majority of material entities, feature a resonant frequency; a frequency at which the amplitude of their oscillations becomes maximum. As with a bell, once a force is applied, the microbubble volumetrically oscillates, thus producing a wave at its resonance frequency. This frequency can be estimated as follows for a free gas bubble [267]:

1 $${\text{f}}_{0} \approx {\text{6}}.{\text{5}}/{\text{D}}$$

where *f*_*τ*_ denotes the resonant frequency and *D* represents the bubble's size. As a result, researchers can deduce that a 2.5 µm gas bubble will resonate at around 2.6 MHz, the frequency of a standard diagnostic US. Microbubbles of free gas are naturally unstable, so they dissolve virtually rapidly. Thus, clinical diagnostic US contrast agents are often composed of an albumin or lipid shell surrounded by a gas core diffusing gradually, which increases consistency and enables effective clinical image analysis [24].

Table 2 summarizes the ultrasonic contrast agents that have been approved for clinical use and their composition.

**Table 2:** US contrast agents with clinical approval

Contrast agent	Shell content	Diameter range (µm)	Type of utilized gas	Creator	Place approved
Definity® [268]	Octafluoropropane (C_3_F_8_)	1.1–3.3	Lipid	Lantheus Medical Imaging	Canada and US
SonoVue® [269]	Sulfur Hexafluoride (SF_6_)	2.5	Lipid	Bracco Int	Europe
Optison™ [268]	Octafluoropropane (C_3_F_8_)	2.2–4.5	Albumin	GE Healthcare	US
Sonazoid® [270]	Perfluorobutane (C_4_F_10_)	1.9–2.4	Lipid	Daiichi Pharmaceutical Co	Japan
BR-55 [271]	Perfluorobutane (C_4_F_10_)	1.5	Phospholipid/lipo-peptide	BRACCO	Clinical trials in Europe
BR-14 [272]	Perfluorobutane (C_4_F_10_)	2.0–2.5	Phospholipid	BRACCO	Clinical trials in Europe

The Mechanical Index (MI) is a parameter used in clinical diagnostic US imaging to indicate the possibility for mechanical damage caused by inertial cavitation in the presence of an ultrasonic contrast agent. It is defined as follows:

2 $${\text{MI}} = {\text{p}}^{ - } /\surd {\text{ f}}_{{\text{c}}}$$

where *f*_*c*_ indicates the center frequency in MHz, and *p*^−^ is the peak-negative acoustic pressure in-situ recorded in MPa. An MI of 0.3 to 0.7 is recognized as relatively secure because there is a high chance of serious harm to the digestive system or neonatal lung tissue, and a MI of > 0.7 carries a greater hazard of inertial cavitation of the US-based contrast agents, and also a scientific vulnerability of cavitation creation without US contrast media. As a result, researchers strive to operate at MI ≤ 0.2 in all of their projects, guaranteeing that there is no harmful and presently unmanageable inertial cavitation, thus enabling a quicker transition from laboratories to clinical trials [273].

To be capable of generating localized sonoporation, the important first step was to try to start regulating the positioning of the microbubbles [274]. On top of an inverted microscope, a custom-designed experimental apparatus with a 2.2 MHz ultrasonic transducer, a manufactured 200-µm capillary, and a high-speed camera was established. The Definity® US contrast agent was employed in combination with the continuous-wave US with center frequencies of 2-MHz and 7-MHz and peak-to-peak acoustical pressures of 20 kPa. The microbubbles were shown to influence and attach to each other after insonation, generating tiny spherical clusters [275, 276]. Within a few seconds, these clusters of 1–2 thousand microbubbles would appear, positioned 1/4 λ off from each other. Once the engaged microbubbles continued to oscillate in phase, the clusters were attracted to each other, generating progressively greater clusters. Clusters could be directed to the membrane wall as the acoustic pressure is increased. These findings suggested that microbubbles could be gathered in certain areas and radiated forward into a vascular wall when required [275, 276].

## Combination of microbubbles and nanoparticles

The US has traditionally been utilized for image processing and diagnostics [277]. Recent technical advancements, particularly in nanoscience, have created unique possibilities for the US to be used in modern medical interventions, such as in situ US-triggered medication manufacturing and minimally aggressive surgical intervention [43, 278, 279]. Liver, prostate, breast, and cancer elimination, uterine fibroid ablation, cataract removal, surgical tissue cutting, phacoemulsification, transdermal drug delivery, and bone fracture therapy have all been proven to be successful using the therapeutic US [280]. In particular, US-assisted medication delivery has gotten a lot of interest in recent years because it allows for spatially limited delivery of therapeutic substances into target locations like tumors [281, 282]. The incorporation of the US and nano-based drug delivery platforms overcomes several major drawbacks of traditional drug delivery systems, such as:

- Inadequate accumulation and uptake of nanostructures by cells [283];
- Limited percentage of medication delivered or released from nanostructures [284];
- Specific targeted delivery of drug-carrying nanomaterials.

Moreover, the use of US in conjunction with nanostructures can increase drug delivery effectiveness and minimize negative impacts by allowing drug-carrying particulates to pass through physiological barriers more easily—a primary aim for modern drug delivery systems [285, 286]. The blood vessels endothelial lining, tight layers of the epithelial cells, targeted tissues endothelium, tissue interstitium, the cells plasma membrane, dissemination through the cytoplasm, and eventual entry into the nucleus via nuclear membrane (if applicable) are all examples of physiological barriers. Furthermore, the BBB is a significant challenge to drug and/or nanoparticle entry into the brain, which could be addressed with the application of US [287, 288].

Systematic injection of chemotherapeutic drugs encapsulated within nanostructures could be used to promote targeted delivery in cancer [289]. They have the potential to increase the efficacy and precision of medication delivery, allowing for tailored medication delivery. Nanostructures can also be coated with substances that identify and bind to cancerous cells to increase targeting. The aberrantly overexpressed receptors of malignancies are the most often used tumor-specific moieties for targeting. Epidermal growth factor receptor (EGFR), human epidermal growth factor receptor 2 (HER2), endothelial growth factor receptor (VEGFR), and folate receptor (FR) are examples of these receptors [290]. Encapsulating therapeutic medication molecules in nanostructures can increase bioavailability, biodistribution, and internalization into targeted cells. Despite recent advances in nanoscience, such as the surface modification of nanomaterials with the mentioned targeting molecules, only about 1% of nanostructures concentrate in tumors [291]. As a result, a successful therapeutic plan for malignant tumors is still challenging. Various physiological obstacles in the tumor structure could demonstrate the limited targeting efficacy [292]. The first challenge for nanomaterials is that they have a significant risk of being removed by blood circulation shortly following intravenous injection, even before they enter the tumor microenvironment. It is also possible because nanomaterials are opsonized with blood proteins, which are then detected by cells of the mononuclear phagocyte system (MPS) and eliminated from the circulatory system. Nanostructure populations that escape MPS removal should diffuse out of the circulatory system. Nanostructures should efficiently concentrate on the endothelial lining toward the tumour microenvironment while in circulation. The second hurdle for nanomaterials is the efficacious extravasation of nanoparticles across the tumor microenvironment. When contrasted to healthy tissues, tumor tissue has a distinctive structure. The tumor configuration frequently has irregular vasculature, overexpression, and a high extracellular matrix density (ECM). The ineffective transport of nanostructures into tumors is mostly due to aberrant tumor characteristics. The ECM of tumors is made up of a crosslinked gel-like framework composed of collagen and elastin fibers, proteoglycans, and hyaluronic acid. The tumor's extremely developed and overexpressed ECM causes severe resistance to therapeutic nanostructure transport via the interstitium. Aside from that, increased interstitial fluid pressure (IFP), which is caused by accelerated cellular proliferation in a small region, high tumor vascular permeability, and the lack of a lymphatic drainage system reduces the force required for nanostructures to infiltrate tumors. These circumstances further prevent nanomaterials from being delivered and distributed evenly throughout the tumor mass [293, 294]. More significantly, the perfusion of the vasculature within a tumor varies greatly, leaving multiple locations with inadequate vascular perfusion and insufficient blood flow. The condition definitely enhances the distance that nanostructures must travel to access targeted cells, resulting in therapeutic medication release and nanoparticle delivery that is excessively far away from the tumor and its microenvironment. All of the tumor's different pathological characteristics significantly impair nanomaterial delivery, diffusion, and accumulation uniformly distributed into the tumor, leading to the treatment's ineffective anticancer influence. The condition definitely enhances the distance that nanostructures must travel to access targeted cells, resulting in nanoparticle delivery and therapeutic medication release that is excessively far away from the tumor and its microenvironment. All of the tumor's different pathological characteristics significantly impair nanomaterial delivery, accumulation, and diffusion uniformly distributed into the tumor, leading to the treatment's ineffective anticancer influence [293, 294].

To address the aforementioned obstacles, a methodology that aids in accurate medication delivery at a tumor location and enhances nanostructure penetrating into tumor mass is required. US-assisted drug-loaded nanomaterial delivery overcomes the restrictions mentioned above by improving nanoparticle concentration and absorption by cells and also triggering the release of the drug specifically at the targeted location [43, 295, 296]. These impacts are produced by a variety of mechanisms such as sonoporosis, cavitation, and hyperthermia, which happen when ultrasonic radiation interacts with cells and nanostructures at the same time. As a result, the application of the US provides the ability to enhance therapeutic targeting, potentially lowering the systemic amount of medicine necessary for effective therapy. As a consequence, US-assisted medication delivery has the potential to lessen total therapy adverse effects such as medication toxic effects and non-targeted distribution [297]. In addition to the utilization of US-assisted medication delivery, several nano-based materials, including MCM-41-SO3H, aryl-14-H-dibenzo[a,j]xanthenes, and tri-and tetrasubstituted imidazoles by nanosized MCM-41-SO, were developed using ultrasonic irradiation and a green approach [298–300].

## Conclusions

The functional value of microbubbles continues to develop through biomedical implementations as innovative formulations and approaches emerge. Microbubbles have a specific set of US reactions that make them valuable in contrasting US-based imaging processes, molecular expression recognition, and drug targeting in particular tissue areas. Developments in our knowledge of basic physical and chemical characteristics have contributed to rapid progress in the manufacture of innovative constructions, including the development of multilayer polyelectrolyte, bimodal brushes, nanoparticle-micro-bubble combinations, and surface compartmentalization.

Nonetheless, standardizing microbubble-assisted focused-US variables and implementing proper safety criteria are critical to improve our knowledge of the consequences and future clinical translation. The various permutations of microbubble, US, and physiological characteristics make this aim difficult to achieve. The absence of optimal and commonly accepted variables in microbubble formulations, dose measurements, experimental animals, and histopathological and behavior patterns assessments for safe operation have hampered productive overlap in previous investigations. Chemical characterization, quality management, and toxicity studies become more important as the microbubble-assisted delivery method becomes more complicated, possibly hindering clinical translation. Further progress in microbubble preparations will improve the functionally applicable platforms as an emerging drug delivery system to combat a wide range of prevalence disorders, and microbubbles with excellent properties will promote new and promising hope for their medical applications, particularly for therapeutic purposes.

## Data Availability

All the data and materials supporting the conclusions were included in the main paper.

## Abbreviations

US: Ultrasound
BBB: Blood–brain barrier
PCDs: Passive cavitation detectors
GDNF: Glial cell line-derived neurotrophic factor
MPTP: 1-Methyl-4-phenyl-1,2,3,6-tetrahydropyridine
BDNF: Brain-derived factor
l-Dopa: Levodopa
PEG: Polyethylene glycol
a-syn: Anti-alpha synuclein
DG: Dentate gyrus
PEG-PLA: Poly(ethylene glycol)-poly(lactic acid)
tPA: Tissue plasminogen activator
AAA: Abdominal aortic aneurysm
PESDA: Perfluorocarbon-exposed sonicated dextrose albumin
pCAT: Plasmid chloramphenicol acetyltransferase
aFGF: Acid fibroblast growth factor
PLGA: Polylactide-co-glycolide
RSG: Rosiglitazone
shRNA: Short-haired RNA
CTGF: Connective tissue growth factor
PTX: Paclitaxel
mAb: Monoclonal antibody
EGFR: Epidermal growth factor receptor
NF-B: Nuclear factor-B
MI: Mechanical Index
PRF: Pulse repetition frequency
HER2: Human epidermal growth factor receptor 2
VEGFR: Endothelial growth factor receptor
FR: Folate receptor
MPS: Mononuclear phagocyte system
ECM: Extracellular matrix
IFP: Interstitial fluid pressure
